# Dataset of active avoidance in Wistar-Kyoto and Sprague Dawley rats: Experimental data and reinforcement learning model code and output

**DOI:** 10.1016/j.dib.2020.106074

**Published:** 2020-07-25

**Authors:** John Palmieri, Kevin M. Spiegler, Kevin C.H. Pang, Catherine E. Myers

**Affiliations:** a Rutgers New Jersey Medical School, Rutgers Biomedical Health Sciences, 185 South Orange Avenue, Newark, NJ 07103, USA; b Rutgers School of Graduate Studies, Rutgers Biomedical Health Sciences, 185 South Orange Avenue, Newark, NJ 07103, USA; c Department of Veterans Affairs, New Jersey VA Health Care System, 385 Tremont Avenue, East Orange, NJ 07018, USA; d Department of Pharmacology, Physiology, and Neuroscience, Rutgers Biomedical Health Sciences, 185 South Orange Avenue, Newark, NJ 07103, USA

**Keywords:** Avoidance learning, Reinforcement learning, Neurosciences, Computational modelling, Computational biology, Strain differences, Wistar Kyoto rat

## Abstract

Data were collected from 40 Wistar-Kyoto (WKY) and 40 Sprague Dawley (SD) rats during an active escape-avoidance experiment. Footshock could be avoided by pressing a lever during a danger period prior to onset of shock. If avoidance did not occur, a series of footshocks was administered, and the rat could press a lever to escape (terminate shocks). For each animal, data were simplified to the presence or absence of lever press and stimuli in each 12-second time frame. Using the pre-processed dataset, a reinforcement learning (RL) model, based on an actor-critic architecture, was utilized to estimate several different model parameters that best characterized each rat's behaviour during the experiment. Once individual model parameters were determined for all 80 rats, behavioural recovery simulations were run using the RL model with each animal's “best-fit” parameters; the simulated behaviour generated avoidance data (percent of trials avoided during a given experimental session) that could be compared across simulated rats, as is customarily done with empirical data. The datasets representing both the experimental data and the model-generated data can be interpreted in various ways to gain further insight into rat behaviour during avoidance and escape learning. Furthermore, the estimated parameters for each individual rat can be compared across groups. Thus, possible between-strain differences in model parameters can be detected, which might provide insights into strain differences in learning. The software implementing the RL model can also be applied to or serve as a template for other experiments involving acquisition learning.

**Reference for Co-Submission:** K.M. Spiegler, J. Palmieri, K.C.H. Pang, C.E. Myers, A reinforcement-learning model of active avoidance behavior: Differences between Sprague-Dawley and Wistar-Kyoto rats. Behav. Brain Res. (2020 Jun 22[epub ahead of print])  doi: 10.1016/j.bbr.2020.112784

**Specifications table**

**Table:** 

**Subject**	Behavioral Neuroscience
**Specific subject area**	Avoidance learning: experimental data and computational modelling in two rat strains
**Type of data**	Empirical dataset (preprocessed animal data)
	Software (code for parameter estimation and for behavioural recovery simulations)
	Dataset generated by behavioural recovery simulations (“simulated data”)
**How data were acquired**	Empirical data: Rats were trained in standard operant chambers with response levers, visual and acoustic stimuli, and grid floors capable of delivering scrambled footshock (Coulbourn Instruments). Graphic State Notation (Version 3, Coulbourn Instruments) software was used to control stimuli/shock and record lever press activity.
	Model data: Custom software (programs written in C, provided as part of this dataset) was used to implement the parameter estimation, and the behavioural simulations.
**Data format**	Cleaned (pre-processed)
	Analysed
**Parameters for data collection**	Experimental data collection was performed in cages designed for footshock acquisition studies. Several conditions (time of training sessions, animal housing, animal handling by same individual) were controlled for in the experimental design.
	For modelling data, custom code implementing an RL model was used to determine individual parameters for each rat related to avoidance acquisition learning.
**Description of data collection**	Empirical data were transformed from the raw data, where presence or absence of lever press and stimuli was noted for each 12-second time segment.
	For parameter estimation, the (preprocessed) empirical data from each rat were fed into the parameter estimation software, to determine a “best-fit” parameter configuration for each rat.
	For behavioural simulation, the best-fit parameters for each rat were used to create a “simulated rat,” which was then trained on 12 sessions of 25 trials of lever press avoidance to determine percent avoidance on each trial.
**Data source location**	VA New Jersey Health Care System
	Medical Research Service
	385 Tremont Avenue, Mail Stop 15A
	East Orange, NJ 07018 USA
	Latitude and Longitude: 40.752396, -74.237254
**Data accessibility**	Repository name: Mendeley Data
	Data identification number: 10.17632/d6ybdxzkwz.4
	Direct URL to data: https://data.mendeley.com/datasets/d6ybdxzkwz/4
**Related research article**	K.M. Spiegler, J. Palmieri, K.C.H. Pang, C.E. Myers, A reinforcement-learning model of active avoidance behavior: Differences between Sprague-Dawley and Wistar-Kyoto rats. Behav. Brain Res. (2020 Jun 22[epub ahead of print]) doi: 10.1016/j.bbr.2020.112784

**Value of the Data**

• These data represent a fairly large sample from two rat strains (*n* = 40 each) trained on an operant escape/avoidance task; the empirical data can be used to investigate between-strain as well as individual differences in how rats learn first to escape from (terminate) an aversive stimulus and then avoid (avert) that aversive stimulus.
• Researchers interested in learning theory, avoidance learning, or strain differences can benefit from this dataset, by applying their own analysis and/or computational modelling techniques to the empirical data; researchers interested in avoidance learning can apply and/or adapt the model estimation software and the behavioural simulation code to their own empirical data.
• These data and the computational model provided can be repurposed for different behavioural learning protocols. The model code can serve as a template for others using actor-critic models to distinguish differences between two populations (either animal or human) and gain insight to various diseases and pathological mechanisms.

## Data description

Pre-processed Empirical Data (S09.csv, S10.csv, etc.): Presence/Absence of lever presses and stimuli for each rat (SD rats S09-S48 and WKY rats W09-W48), discretized to 12-second time periods

**Fig. 1:**
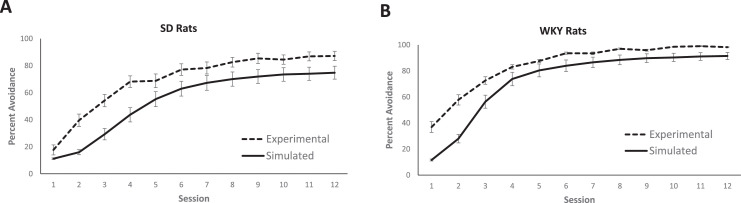
**Avoidance acquisition behavior in (A) Sprague-Dawley (SD) and (B) Wistar-Kyoto (WKY) rats.** In each graph, the dotted line is based on the empirical data in this dataset; the solid line is based on the results of simulations using the extracted parameters included in this dataset, using the parameter estimation code and the behavioral simulation code. Avoidance is shown as a percentage of trials in each session during which an avoidance response occurred. Error bars represent the standard error.

Binary data for the presence/absence of lever presses and stimuli occurring during each 12-second timestep are presented for each rat. The 12-second timesteps are represented as row headings whereas session number and the trials within each session are represented as column headings. The 5 columns following the information on session and trial # correspond to the presence (1) or absence (0) of five different stimuli: danger signal, safety signal, shock, chamber, homecage. For example, if the rat is in the experimental chamber and experiencing a danger period, then the cells for “danger” and “chamber” will be marked as a “1” whereas the 3 other stimuli will be marked as “0”. Entries within the final column correspond to the presence (1) or absence (0) of a lever presses during that given 12-second timestep. Experimental avoidance acquisition data generated from the pre-processed empirical data can be seen in Fig. 1A-B for SD and WKY rats respectively.

1. Supplementary Data #1 (called “listfile.txt”): Input for the Parameter Estimation Model - A list of rats for which parameters will be optimized

The “listfile.txt” is a text formatted document containing a list of the 80 rats for which the parameter-fitting is being conducted. For example, rats “S09-S48” are listed for the 40 SD rats while “W09-W48” are listed for the WKY rats. To run only certain rats, one could simply delete rat names from the “listfile.txt”, adding additional flexibility to how the model-fitting code can be operated.

  ○ Note that the “Pre-processed Empirical Data” (above section) are also an input into the parameter estimation model.
2. Supplementary Data #2 (called “estimated_parms_ModelC.csv”): Output of the Parameter Estimation Model – Learning parameters, action policy evaluations (M-values), and state evaluations (V-values) for each rat.

Parameters generated from the parameter estimation software for each rat (n=80). Rats are presented as row headings whereas the parameters are presented as 7 column headings. A rat name starting with an “S” refers to a Sprague Dawley (SD) rat whereas a rat name starting with a “W” refers to a Wistar-Kyoto (WKY) rat. For example, S15 is an SD rat whereas W29 is a WKY rat. The 7 parameters are reported as rational numbers. A list of parameters is reported below:

• *α-* learning rate in critic
• *β-* Learning rate in actor
• *Β –* Exploitation /Exploration parameter
• *R_shock –_* reinforcement value of shock
• *R_press –_* reinforcement value of press (held constant in the “winning” model, Model C)
• *P –* perseveration
• *γ* – discount factor

**Fig. 2:**
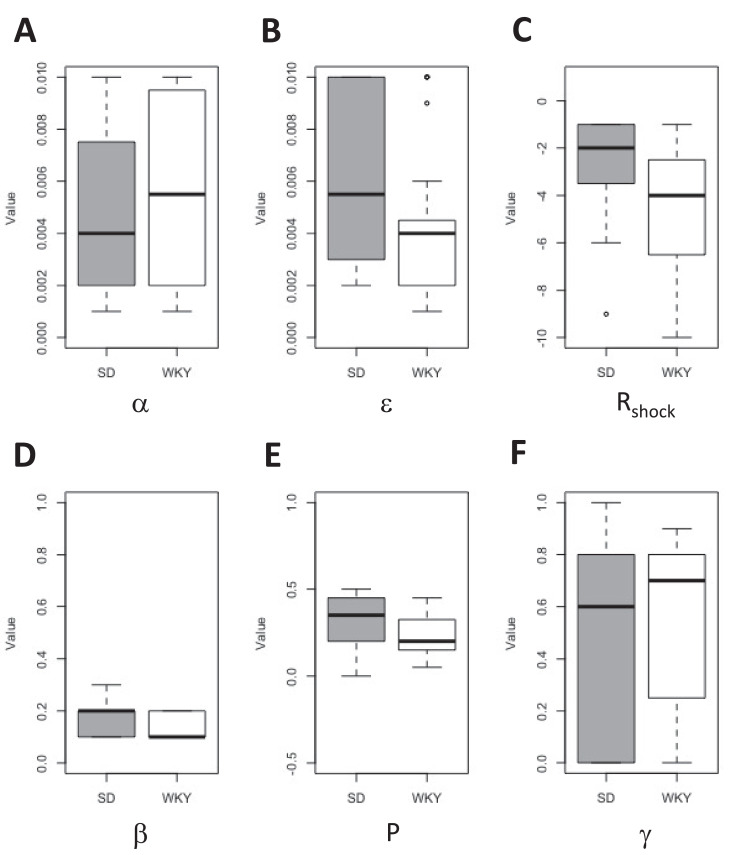
**Distributions of model parameter estimations in SD and WKY rats.** Estimated parameters were derived for each rat, based on the empirical data in this dataset, using the parameter estimation code with 6 free parameters: (A) learning rate in the critic (*α*); (B) learning rate in the actor (*ε*); (C) reinforcement value of shock (*R_shock_*); (D) exploitation/exploration (*β*); (E) perseveration (P); (F) discount factor (*γ*). The median value for each parameter is depicted as a solid horizontal line; boxes indicate interquartile range (Q_1_ - Q_3_) and whiskers indicate range excluding outliers (depicted as open dots) defined as >1.5 IQR beyond Q_1_ or Q_3_.

*α* refers to the learning rate of the critic, while *ε* refers to the learning rat in the actor. *Β* refers to an exploration/exploitation parameter whereby higher value of *Β* reflect tendency to exploit previously successful strategies and low values indicate tendency to occasionally explore new ones. *R_shock_* refers to the rat's subjective experience of the shock whereas larger negative values refer to shocks that are perceived to be more unpleasant. R_press_ refers to the rat's subjective effort or cost of pressing the lever. *P* (perseveration*)* refers to the rat's tendency to repeat previous responses regardless of their outcomes. Finally, *γ* refers to the rat's discount factor in assessing the value of future rewards/states in decision-making in the present. Larger *γ* values mean that the rat is more “forward-thinking” in assessing the value of future states in decision-making. Box and whisker plots for estimated values of the 6 free parameters can be seen in Fig. 2A-F. For further details of the RL model and parameters, please see Spiegler et al. (2020) [1].

After the 7 parameters, there is a column for “negLLE” (negative log likelihood estimate), a measure of how well the model, using these parameter values, fits the data. Smaller values (closer to 0) reflect better fit.

The next 15 columns include values obtained from the best-fit model for each rat, at the end of the final acquisition session. These include information on action policies (M-values), including tendency to press or not press (other) in the presence of each stimulus. The first 5 columns (“m_OTHER_DS”, “m_OTHER_SS”, “m_OTHER_Sh”, “m_OTHER_chamb”, and “m_OTHER_home”) are m-weights associated with tendency to execute other response (i.e. not lever press) in the presence of danger signal (DS), safety signal (SS), shock (Sh), or while in the experimental chamber (chamb) or home cage (home). The next 5 columns (“m_PRESS_DS”, “m_PRESS_SS”, “m_PRESS_Sh”, “m_PRESS_chamb”, and “m_PRESS_home”) are m-weights associated with tendency to execute lever press in presence of each stimulus. The next 5 columns include information on state evaluations (V-values) in the presence of each stimulus (“V _DS”, “V _SS”, “V _Sh”, “V _chamb”, and “V _home”).

The next 21 columns just provide a record of the ranges and step sizes in explored for each of the 7 parameters described above. For example, the α value was explored from “Amin” (lower bound) to “Amax” (upper bound) in steps of size “Astep.”

The final column records the number of overnight sessions used to represent the rat returning to the home cage after an acquisition session (for Model C, this was set to 500 timesteps for each rat).

3. Supplementary Data #3 (called “parm_listfile.csv”): Input into the Behavioural Simulation Model

Estimated model parameters for each rat that should be simulated by the behavioural recovery program. (These may be values obtained from the parameter estimation software.) Rats are presented as row headings whereas the parameter values are presented as 6 column headings (no R_press_ since it is held constant here). A rat name starting with an “S” refers to a Sprague Dawley (SD) rat whereas a rat name starting with a “W” refers to a Wistar-Kyoto (WKY) rat. For example, S15 is an SD rat whereas W29 is a WKY rat. The 6 parameters are reported as rational numbers. See Supplementary Data #2 for more information on the parameters.

This file was used as input to the behavioural simulations, specifying the values of each parameter to be used in simulating each rat.

4. Supplementary Data #4: (S09sum.csv, S10sum.csv, etc.): Example summary files provided for each rat (SD rats S09-S48 and WKY rats W09-W48)

These files are generated by the model estimation program, and summarize the behavioural data for each rat. Each row represents one session; for each trial, the response is scored as “A” if an avoidance occurred, “E” if an escape occurred, or “.” if no lever press occurred. Below that, one row per session records the total anticipatory responses (ARs) occurring during the habituation period, and total responses during the ITI (inter-trial responses, ITRs) following each trial.

These files are then accessed by the behavioural simulation program, allowing comparison of the responses generated by the simulations against the actual rat's behaviour.

5. Supplementary Data #5: Output of Behavioural Simulation Model (S09out.csv, S10out.csv, etc.) – Number of avoidances, escapes, anticipatory responses (ARs), and inter-trial responses (ITRs) for each simulated rat, re-initialized and trained over 100 simulated acquisition experiments.

Each file reports results of 100 simulations of that rat, using estimated parameter files as given by the “parm_listfile.csv” file.

In the first block of rows, trial-by-trial responses are reported. Each run is represented as a row whereas session number and trial number within the given session are represented as the column headings. For example, a column heading of 5_17 represents the 17^th^ trial of the 5^th^ acquisition session. Data are reported first for escapes and avoidances, where a lever press during a danger period is marked as “A” for avoidance, and a lever press during a shock period is marked as “E” for escape. Simulated avoidance acquisition data generated from the behavioural simulation model output can be seen in Fig. 1A-B for SD and WKY rats, respectively.

The next section reports the number of anticipatory responses (ARs), which are lever presses during the habituation periods at the start of each session (one column for each of 12 sessions).

The final section of the file reports the number of ITRs (inter-trial responses), which occur during the safety period (inter-trial-interval) following each trial. For comparison with how empirical data are typically analysed, each inter-trial interval is broken into 3 one-minute periods, and the number of ITRs during that period is reported (e.g. column header 2_3_1_ITRs refers to session 2, trial 3, first minute of inter-trial interval) [2].

## Experimental design, materials and methods

The empirical data were collected from an experiment involving 40 Wistar-Kyoto (WKY) and 40 Sprague Dawley (SD) rats from Envigo (Indianapolis, IN) who arrived at 2.5-3 months of age [1]. Rats were acclimated to housing for 2 weeks. Throughout the experiment, rats were housed in a 12-hour on/12-hour off light cycle starting at 7am and were given rat chow and water *ad libitum*. Rats then underwent 12 sessions of active avoidance acquisition, 3 times a week with at least 48 h between sessions. Each session consisted of 25 trials with three possible phases: a danger period (75 dB tone, no shock, maximum of 72 s), a shock period (75 dB tone, shock, maximum of 72 s), and a “safe” inter-trial interval (ITI) period (5 Hz flashing light, no shock, fixed duration of 180 s). During the danger periods, rats could press a lever located in the operant box (Coulbourn Instruments) in order to avoid the following shock period. If a lever press occurred during the danger period (avoidance response), the shock period was skipped and the ITI began. However, if a lever press did not occur during the danger period, then the shock period began (1 mA, 0.5 s duration, 1 shock/3.5 s, maximum 20 shocks). If a lever press occurred during the shock period (escape response), the shock period was terminated and the ITI began. Every session started with a habituation period of 60 s in the experimental chamber (no tone, light, or shock). Upon completion of each session, the rats were removed from the experimental cage and were returned to their home cages (no tone, light, or shock).

The raw data obtained during the trials in the experimental cage were transformed into discrete intervals (“timesteps”) of 12-seconds during which presence or absence of lever presses and stimuli were noted. Lever presses occurring during habituation were labelled as anticipatory responses (ARs) whereas lever presses occurring during ITI periods were labelled as inter-trial responses (ITRs) [1, 2]. The pre-processed data also included timesteps representing inter-session time in the home cage; however, since there was no lever available in the homecage and the rat behaviour was not recorded there, these timesteps were always scored as having no tone, light, shock, or lever press.

The pre-processed data (with the presence/absence of stimuli and lever presses for each timestep) for each rat were then used as input to parameter estimation software. In particular, an actor-critic (AC) model was utilized to determine a set of “best-fit” parameters for each rat that allowed the model to best reproduce that rat's behaviour (minimizing negative log-likelihood estimate, negLLE) [3]. The parameter estimation code essentially runs through every possible combination of parameter values for each rat and records the configuration providing “best-fit” based on minimization of negLLE. Box and whisker plots for all 6 of the free parameters can be seen in Fig. 2A-F. State (V-values) and action (M-values) were recorded for each rat, based on values in the model at the end of training under those best-fit parameters. V-values refer to how each animal evaluates a certain state (such as a shock state, danger state, etc) whereas M-values refer to how each animal evaluates a certain action within a given state (such as a lever press during a shock period or not pressing a lever during a danger period). For additional details on the RL model, see Spiegler et al. (2020), or the documentation accompanying the parameter estimation code [1].

Based on the results from the parameter estimation, behavioural recovery simulations were run. Specifically, the “best-fit” parameters for each rat were used to construct a “simulated rat” that could be run through 12 acquisition sessions, to generate percentage of avoidances for each session; 100 simulations were run for each rat, and the results were averaged across these simulations. Experimental and simulated avoidance acquisition behaviour are depicted in Fig. 1A-B.

Detailed instructions for running and compiling the parameter estimation software and behavioural recovery software are included in Mendeley Data (instructions to use).

Data identification number: 10.17632/d6ybdxzkwz.4

Direct URL to data: https://data.mendeley.com/datasets/d6ybdxzkwz/4

## Ethics statement

All animal procedures were carried out in accordance with the National Institutes of Health *Guide for the Care and Use of Laboratory Animals* and were approved by the Institutional Animal Care and Use Committee at the VA New Jersey Health Care System.

## Declaration of Competing Interest

The authors declare that they have no known competing financial interests or personal relationships which have, or could be perceived to have, influenced the work reported in this article.

## Appendix Supplementary materials

Image, application 1
