# Supplementary material for: Dataset of active avoidance in Wistar-Kyoto and Sprague Dawley rats: Experimental data and reinforcement learning model code and output
Source: Data Brief. 2020 Jul 25;32:106074. doi: 10.1016/j.dib.2020.106074 (PMC7451822; doi:10.1016/j.dib.2020.106074)
Supplement: Supplementary file 1 [file mmc1.zip › Mendeley Data Instructions.docx]

Mendeley Data Instructions

**Title:**

A Reinforcement-Learning Model of Active Avoidance in Wistar-Kyoto and Sprague Dawley Rats: Experimental Dataset with Model Code and Output

**Data Description:**

This study used a reinforcement learning (RL) model to examine strain differences in rats learning an active avoidance task. The empirical data were previously published [1]; preprocessed data are included here. In brief, 40 male Wistar-Kyoto (WKY) and 40 male Sprague Dawley (SD) rats were trained to make an operant lever press response to avoid or escape from aversive footshocks. Responses made during a danger period that preceded shock onset were scored as avoidance responses and averted the shock; responses made during the shock period were scored as escape responses and terminated the shock. Each rat received 12 acquisition sessions of 25 trials each.

For the current study, raw data from the empirical study were preprocessed by discretizing data from each rat (SD rats S09-S48 and WKY rats W09-W48) to 12-second periods (“timesteps”), and noting presence or absence of experimental stimuli (danger signal, safety signal, and/or shock), the context (experimental chamber or home cage), and whether the rat emitted at least one lever press within that timestep. These can be found in the “**ratRL_datafiles_Model_InputOutput**” folder (e.g. S09.csv, S10.csv, etc.).

A reinforcement learning (RL) model was applied to these preprocessed empirical data to estimate model parameters for each rat that best reproduced that rat’s behavior. Code can be found in the “**ratRL_ModelFitting_Code**” folder. The program estimates model parameters and also generates a summary file for each rat (e.g. S09sum.csv, S10sum.csv, etc.) noting whether an escape or avoidance response occurred on each trial.

Given estimated parameter values for each rat, behavioral recovery simulations were conducted, by constructing “simulated rats” based on the parameter values, which were then trained on the avoidance acquisition task. For each simulated rat, results were averaged over 100 runs. Code can be found in the “**ratRL_Simulation_Code**” folder. Example inputs to this simulation are found in the “**ratRL_codedTrials_Sim_InputOutput**” folder, and include both a list of parameter values for each simulated rat (“parm_listfile.csv”) as well as the summary file for each rat (e.g. “S09sum.csv”) generated by the model fitting program.

The results of the parameter estimation can be used to examine differences within and across strains; for example, whether there are differences in the estimated value of a particular parameter, such as the subjective reinforcement value of shock, for SD vs. WKY rats. The results of the behavioral recovery simulations can be analyzed in the same way as empirical data; for example, after averaging across 100 simulation runs to get an average learning curve for each rat, the performance of simulated rats of each strain can be compared to determine if there is a main effect of trial/session (indicating learning), a main effect of strain (e.g. simulated WKY show more avoidance responses than simulated SD), and/or an interaction.

**STEPS TO REPRODUCE**

**Steps to Reproduce the Parameter Estimation (estimated parameter values for each rat):**

1. In the folder titled “ratRL_ModelFitting_Code” open the file called “main.c” and alter the default path to specify full pathname of the folder where the input/output files are located; e.g.

char default_path[STR_LEN] =

"/Users/johnsmith/Desktop/ratRL_datafiles_Model_InputOutput"

This folder should contain one preprocessed datafile for each rat (e.g. “S09.csv” for SD rat S09, etc.) and the supporting file “listfile.txt” with list of rat “names” to process.

The “include.h” file can be modified before compiling to alter granularity of parameter exploration space, by changing stepsize (and/or range) for each estimated parameter.

1. From the command line prompt, set the current working directory to the folder where the C code files are located, compile and run the program, e.g.:

cd /Users/johnsmith/Desktop/ratRL_ModelFitting_Code

gcc main.c ACmodel.c rat_fcns.c -o ratRL_Model

./ratRL_Model

1. Output files include the estimated parameters (“estimated_parms.csv”) and one summary file for each rat (e.g., “S09sum.csv”), placed in the default folder; interim and final parameter estimates also appear in the terminal window.

**Steps to Reproduce the Behavioral Recovery Simulations:**

- - - 1. In the folder titled “ratRL_ Simulation Code” open the file called “main.c” and alter the default path to specify full pathname of the folder where the input/output files are located, e.g.:

char default_path[STR_LEN] =

"/Users/johnsmith/Desktop/ratRL_codedTrials_Sim_InputOutput”;

This folder should contain one summary file for each rat (e.g., S09sum.csv, etc., generated by the model estimation program) and a file entitled “parm_listfile.csv” specifying parameter values to simulate each rat.

1. From the command line prompt, set the current working directory to the folder where the C code files are located, then compile and run the program, e.g.:

cd /Users/johnsmith/Desktop/ratRL_Simulation_Code

gcc main.c ACmodel.c file_io.c utilities.c -o ratRL_Sim

./ratRL_Sim

1. Output files will be stored in the default folder (one for each simulated rat (e.g. S09out.csv, S10out.csv, etc.). The files include one row for each of 100 simulations of that rat, coding each trial by response (A=avoidance, E=escape, .=no response). Below the responses, for each simulation appear the number of anticipatory responses (ARs) that occurred during the habituation period at the start of each of the 12 sessions, and then the number of responses during the inter-trial interval (ITI) with each ITI broken into three one-minute intervals; thus; 1_2_1_ITRs refers to session 1, trial 2, first minute of the ITR. Summary results for each rat (grand mean of avoidance responses per session and overall percent of trials on which the simulation behavior A/E/no response matched the rat’s behavior), averaged over the 100 simulation runs, appear in the terminal window.

References:

[1] KM Spiegler, IM Smith, KC Pang. Danger and safety signals independently influence persistent pathological avoidance in anxiety-vulnerable Wistar Kyoto rats: A role for impaired configural learning in anxiety vulnerability, Behav.Brain Res. 356 (2019) 78-88.
